# Action of Oil of Turpentine, Opium, &c., on the Nervous System

**Published:** 1831

**Authors:** 


12. *Action of Oil of Turpentine, Opium, &c. on the Nervous System.*—M. Flourens has lately detailed a series of experiments made by him, on the action of essential oil of turpentine, opium, and alcohol, when applied to different parts of the brain. The operation was performed on rabbits, the cranium and dura mater having been previously removed: in all the experiments he took care to renew the substances as soon as they disappeared from the surface of the organ, by absorption or evaporation. The oil of turpentine applied to the lobes of the brain, produced an agitation, with occasional intervals of repose. Sometimes the animal leaped forward, and at others turned round, in a spiral direction. During the paroxysm, the animal appeared in a state of furious madness, and neither saw nor heard but in the intervals of repose; the faculties of sight and hearing appeared uninjured. On applying the turpentine to the cerebellum, a strong tendency to run and leap, was observed. The effects of alcohol were similar, but less violent, never producing the circumgyration. Liquid opium applied to the lobes of the brain, quickly induced torpor, which continued for some time. Opium applied to the cerebellum, destroyed the equilibrium of the body, and the animal could move only on its abdomen.—*Ib.*

